# Monitoring Utilization Trends of Glucose-Lowering Drugs for Type 2 Diabetes in Older Adults by Frailty Status

**DOI:** 10.1093/geroni/igab046.804

**Published:** 2021-12-17

**Authors:** Chandrasekar Gopalakrishnan, Dae Kim, Alexander Kutz, Elisabetta Patorno

**Affiliations:** 1 Brigham and Women's Hospital and Harvard Medical School, Boston, Massachusetts, United States; 2 Hebrew SeniorLife, Boston, Massachusetts, United States; 3 Brigham and Women's Hospital, Harvard Medical School, Boston, Massachusetts, United States

## Abstract

Using Medicare fee-for-service data from 2013-17, we identified a cohort of patients with type 2 diabetes (T2D) who initiated a glucose-lowering drug (mean [SD] age, 74.8 (6.9) years). Amongst frail patients (CFI≥0.20), metformin use remained stable from 29.1% to 29.4%, whereas sulfonylureas (25.8% to 22.1%) and insulin (21.2% to 19.0%) use declined. Amongst non-frail patients (CFI <0.20), metformin (35.3% to 33.1%) and sulfonylurea (26.2% to 22.2%) use decreased whereas insulin (11.7% to 10.6%) use remained stable. DPP-4i and glitazones use remained stable whereas the use of newer agents such as SGLT-2i and GLP-1 RA increased steadily over the study period in both frail and non-frail patients, though their use remains low ( <8%). In conclusion, sulfonylureas and insulin accounted for about one-third of initiated glucose-lowering medications and were more frequently used by frail patients, though their use declined steadily over time with the availability of newer agents.

